# A Rare Case

**Published:** 1886-03

**Authors:** C. F. Paine

**Affiliations:** Comanche, Texas


### A RARE CASE.

COMANCHE, TEXAS, Feb. 2, 1886.

ED. DANIEL'S MEDICAL JOURNAL:

THE following case occurred in my practice December 30, 1885: Mr. R. was confined of her fourth child, breech presentation. Labor was soon terminated without any unusual trouble. Upon examination of the child, however, which was a large, well developed male, I found the right arm off, just below the elbow. A small cartilaginous appendage, somewhat resembling the thumb or finger, projected from the stump.

No clue to the cause.

Yours truly, C. F. PAINE.

